# Increased Endocytosis of Cadmium-Metallothionein through the 24p3 Receptor in an In Vivo Model with Reduced Proximal Tubular Activity

**DOI:** 10.3390/ijms22147262

**Published:** 2021-07-06

**Authors:** Itzel Pamela Zavala-Guevara, Manolo Sibael Ortega-Romero, Juana Narváez-Morales, Tania Libertad Jacobo-Estrada, Wing-Kee Lee, Laura Arreola-Mendoza, Frank Thévenod, Olivier Christophe Barbier

**Affiliations:** 1 Departamento de Toxicología, Centro de Investigación y de Estudios Avanzados del Instituto Politécnico Nacional, Av. Instituto Politécnico Nacional 2508, Col. San Pedro Zacatenco, México CP 07360, Mexico; pamela0191@hotmail.com (I.P.Z.-G.); rom_0@hotmail.com (M.S.O.-R.); juana_narvaez@hotmail.com (J.N.-M.); 2 Departamento de Biociencias e Ingeniería, Centro Interdisciplinario de Investigaciones y Estudios Sobre Medio Ambiente y Desarrollo, Instituto Politécnico Nacional, 30 de Junio de 1520 s/n, Col. Barrio la Laguna Ticomán, México CP 07340, Mexico; tjacoboe@ipn.mx; 3 Department of Physiology, Pathophysiology and Toxicology and ZBAF (Center for Biomedical Education and Research), Faculty of Health-School of Medicine, Witten/Herdecke University, 58448 Witten, Germany; Wing-Kee.Lee@uni-wh.de (W.-K.L.); frank.thevenod@uni-wh.de (F.T.); 4 Physiology and Pathophysiology of Cells and Membranes, Medical School OWL, Bielefeld University, 33615 Bielefeld, Germany

**Keywords:** cadmium nephrotoxicity, gentamicin-induced nephrotoxicity, metallothionein uptake, distal tubule

## Abstract

Background: The proximal tubule (PT) is the major target of cadmium (Cd^2+^) nephrotoxicity. Current dogma postulates that Cd^2+^ complexed to metallothionein (MT) (CdMT) is taken up through receptor-mediated endocytosis (RME) via the PT receptor megalin:cubilin, which is the predominant pathway for reuptake of filtered proteins in the kidney. Nevertheless, there is evidence that the distal parts of the nephron are also sensitive to damage induced by Cd^2+^. In rodent kidneys, another receptor for protein endocytosis, the 24p3 receptor (24p3R), is exclusively expressed in the apical membranes of distal tubules (DT) and collecting ducts (CD). Cell culture studies have demonstrated that RME and toxicity of CdMT and other (metal ion)–protein complexes in DT and CD cells is mediated by 24p3R. In this study, we evaluated the uptake of labeled CdMT complex through 24p3R after acute kidney injury (AKI) induced by gentamicin (GM) administration that disrupts PT function. Subcutaneous administration of GM at 10 mg/kg/day for seven days did not alter the structural and functional integrity of the kidney’s filtration barrier. However, because of PT injury, the concentration of the renal biomarker Kim-1 increased. When CdMT complex coupled to FITC was administered intravenously, both uptake of the CdMT complex and 24p3R expression in DT increased and also colocalized after PT injury induced by GM. Although megalin decreased in PT after GM administration, urinary protein excretion was not changed, which suggests that the increased levels of 24p3R in the distal nephron could be acting as a compensatory mechanism for protein uptake. Altogether, these results suggest that PT damage increases the uptake of the CdMT complex through 24p3R in DT (and possibly CD) and compensate for protein losses associated with AKI.

## 1. Introduction

Cadmium (Cd^2+^) is a transition metal that is released into the environment by natural and anthropogenic activities. Since Cd^2+^ is not degraded in the environment, the risk of human exposure is constantly increasing, posing an important threat to human health because it can damage multiple organs, mainly the kidneys. Environmental exposure to Cd^2+^ occurs mostly through contaminated dietary sources, cigarette smoking, industrial fumes, and dust. Therefore, the main routes of exposure to Cd^2+^ are oral and inhalation [1].

Following absorption, Cd^2+^ in the circulation tends to concentrate in blood cells (erythrocytes and leukocytes) and <10% remains in the plasma where Cd^2+^ binds mainly to albumin and other proteins and peptides such as glutathione (GSH), metallothionein (MT), β2-microglobulin, α1-microglobulin, or transferrin (Tf) [2,3]. Once Cd^2+^ is transported to the liver, it is taken up by the hepatocytes [4] or Kupffer cells [5], in which it induces the synthesis of detoxifying proteins, such as MT. MT is a cysteine-rich metal-binding protein that chelates up to 7 Cd^2+^ ions due to the high affinity that Cd^2+^ has for the sulfhydryl groups of this protein (*K_D_* for Cd^2+^~10^−14^ mol/L), thus reducing its toxic effects [6]. During chronic exposure to low Cd^2+^ concentrations, it is assumed that due to the cellular damage induced by Cd^2+^, or by the cellular turnover of hepatocytes, Cd^2+^ as CdMT complex is released into the bloodstream, being the predominant form in which it is found in the circulation and redistributed to the kidney [7,8].

In the kidney, the PT is the major target of Cd^2+^ toxic effects. Current dogma postulates that once the CdMT complex is filtered by the glomerulus (due to its low molecular weight (~7 kDa)), it is reabsorbed by the epithelial cells of the proximal segment (mainly the S1 and S2 segments) through receptor-mediated endocytosis (RME) via the megalin:cubilin receptor [9]. The low-affinity and high-capacity multi-ligand receptor megalin:cubilin is expressed in the brush border membrane (BBM) of the PT where it binds and internalizes a large number of filtered proteins, including MT [10,11]. Following RME, the CdMT complex is delivered to lysosomes [12,13] where the protein MT is degraded, and free Cd^2+^ is transported to the cytosol through divalent metal transporter 1 (DMT1) where it causes toxicity [14,15,16]. Cd^2+^ accumulation in the PT is promoted by the absence of an efflux pathway for delivery of cytosolic Cd^2+^ into the extracellular fluid or blood plasma because ferroportin 1 (FPN1) that is expressed at the basolateral cell side of PT cells does not transport Cd^2+^ [17,18]. Chronic exposure to Cd^2+^ results in its accumulation in the kidney where it may cause damage, fibrosis or failure [19]. In contrast, acute or subchronic Cd^2+^ exposure is associated with proteinuria, aminoaciduria, glucosuria and phosphaturia [20].

Although the current dogma also assumes that distal tubules (DT) and collecting ducts (CD) cells do not carry out RME [21], it has been demonstrated that physiologically a small but significant proportion of filtered proteins are reabsorbed by the distal segments of the nephron [22]. Hence some proteins may bypass reuptake in the PT as a consequence of their low affinity to megalin or due to limited uptake capacity of the system (e.g., following glomerular or hereditary/ acquired PT damage, i.e., a renal Fanconi syndrome, and ensuing proteinuria) [23,24,25]. Consequently, following glomerular or PT damage, the later segments could become more relevant for uptake, which could make them more susceptible to complexes of proteins with xenobiotics, such as Cd^2+^. Indeed, MT has a low affinity to megalin (*K_D_*~100 µM) [26], and the concentration of MT in the ultrafiltrate is only about 0.5–5 nM [27]. This suggests that CdMT may bypass megalin expressed in the PT and other Cd^2+^-protein complexes are more likely bound and internalized by megalin causing Cd^2+^ accumulation and toxicity in the PT [3]. Accordingly, in vitro studies using a rat PT cell line expressing the megalin receptor showed that Cd-β2-microglobulin, Cd-Albumin and Cd-Lipocalin-2 complexes are taken by PT cells and cause toxicity at ultrafiltrate concentrations, and that the CdMT complex is not toxic even at concentrations that exceed by a hundred-fold the physiological MT concentrations found in the ultrafiltrate [28].

Neutrophil gelatinase-associated lipocalin (NGAL (humans), siderocalin or 24p3 (rodents) is a small endogenous protein (~25 kDa) that is upregulated by damaged epithelia exposed to various noxious agents, including the kidneys [29,30]. Lcn2 is a protein that donates Fe to cells through complexation with bacterial and mammalian siderophores [31]. Lcn2 delivers Fe to epithelia through endocytosis and endosomal acidification in a process similar to cellular Tf handling [3] in order to stimulate their growth and differentiation, and promote repair and regeneration of damaged epithelia, e.g., during acute kidney injury (AKI) [32]. Its uptake is mediated by megalin:cubilin in the PT [10,11], but also by a specific high-affinity low-capacity receptor, the NGAL/24p3/Lipocalin-2 receptor (gene name *Slc22a17*) [33], which is expressed apically in the distal nephron, particularly in distal tubules (DT) in the renal cortex, as well as in cortical and medullary collecting ducts (CD) [34,35,36].

In accordance, there is evidence that distal parts of the nephron are also sensitive to damage induced by Cd^2+^, an effect observed in experimental animals and workers exposed to this metal [37,38]. Distal parts of the nephron can reabsorb Cd^2+^ through Fe^2+^ and/or Ca^2+^ transport pathways [3]. And evidence from cell culture experiments shows that uptake of Fe in the distal nephron may involve RME of Fe protein complexes via 24p3R [34,39]. Moreover, when 24p3R is overexpressed in Chinese hamster ovary cells, as well as in a mouse distal convoluted tubule (DCT) cell line that endogenously expresses 24p3R, the receptor internalizes submicromolar concentrations of MT, Tf and albumin. Also, when both cell lines are incubated with the CdMT complex, the cell viability decreases as the concentration of the complex increases [34]. Considering that in the kidney, 24p3R is expressed in DT and CD, but not in PT [34], it is possible that in vivo following PT damage induced by other Cd^2+^-protein complexes, 24p3R in the distal nephron may additionally contribute to endocytosis of CdMT, possibly increasing renal damage.

For this reason, the present study was designed to examine if in an in vivo model of PT injury induced by gentamicin (GM) increased uptake of the CdMT complex by 24p3R occurs in the distal nephron (DT and possibly CD) as a compensatory mechanism. The aminoglycoside GM was used because it is endocytosed by megalin:cubilin and thereby induces structural damage, dysfunction and/or death of PT cells, thus mimicking renal Fanconi syndrome [40,41,42].

## 2. Results

To test our hypothesis, we first performed a dose-response curve with GM to determine the dose at which the aminoglycoside only damaged the PT but the physiological function of the glomerulus and the integrity of the DT (and CD) was preserved. After GM administration, physiological parameters, such as body weight and water intake were not modified (data not shown).

### 2.1. Effect of GM Administration on Glomerular Function

**Figure 1:**
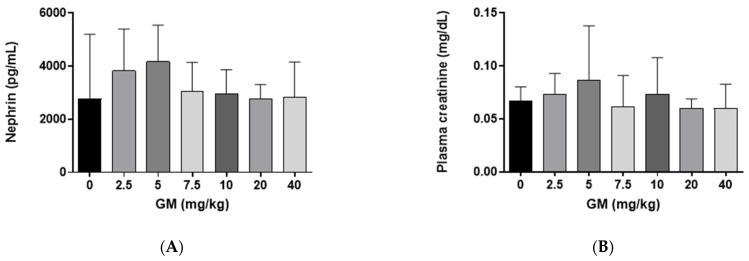
Subacute administration of GM does not modify the glomerular filtration barrier of C57BL/6J mice (**A**) Quantification of nephrin in whole renal tissue. One-way ANOVA, Kruskal Wallis, *p* = 0.7489; (**B**) Plasma creatinine levels. One-way ANOVA, Kruskal Wallis, *p* = 0.6638. Bar graphs show means ± SD, *n* = 5 animals per dose.

Nephrin is a transmembrane protein expressed in glomerular podocytes which, together with endothelial cells and the basement, forms the glomerular filtration barrier that obstructs the filtration of large molecules into the urinary space [43]. The concentration of nephrin in renal tissue after GM administration was quantified to evaluate if GM modified the structural and functional integrity of the kidney’s filtration barrier. Nephrin did not show changes after GM administration when compared with the control group (Figure 1A). Similarly, plasma creatinine did not change between the different GM-exposed groups (Figure 1B).

### 2.2. Effect of GM Administration on Tubular Injury

**Figure 2:**
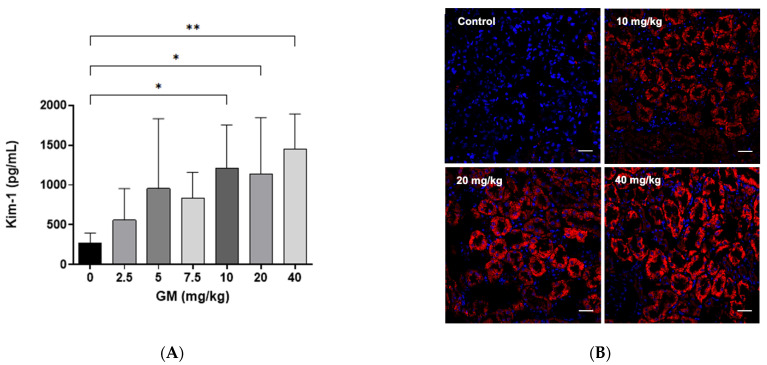
Kim-1 protein increases after GM administration for 7 days. (**A**) Quantification of Kim-1 protein in whole renal tissue using xMAP technology. Bar graph shows means ± SD. *n* = 5 animals per dose. One-way ANOVA was performed *p* = 0.0071. Post-hoc: Dunn test. * *p* < 0.05; ** *p* < 0.005.; (**B**) Representative micrographs of the expression pattern of Kim-1 protein (red fluorescence) in renal tissue after the exposure to 10, 20 and 40 mg of GM/kg/day. The nuclei were stained with 4,6-diamino-2-phenylindole (DAPI, blue). Scale bars represent 50 µm.

To evaluate whether GM administration damaged the PT, protein expression of kidney injury molecule-1 (Kim-1) was measured [41]. As a result of GM exposure, we found that the concentration of Kim-1 measured by Magnetic Luminex assay increased significantly upon the administration of 10, 20 and 40 mg/kg/day of GM when compared with the control group (Figure 2A). Immunolabeling of Kim-1 was not detectable in the kidney tissue from the control group. Moreover, Kim-1 protein expression in renal cortex, increased significantly in PT at 10–40 mg/kg/day of GM administration, when detected by immunofluorescence microscopy (Figure 2B).

**Figure 3:**
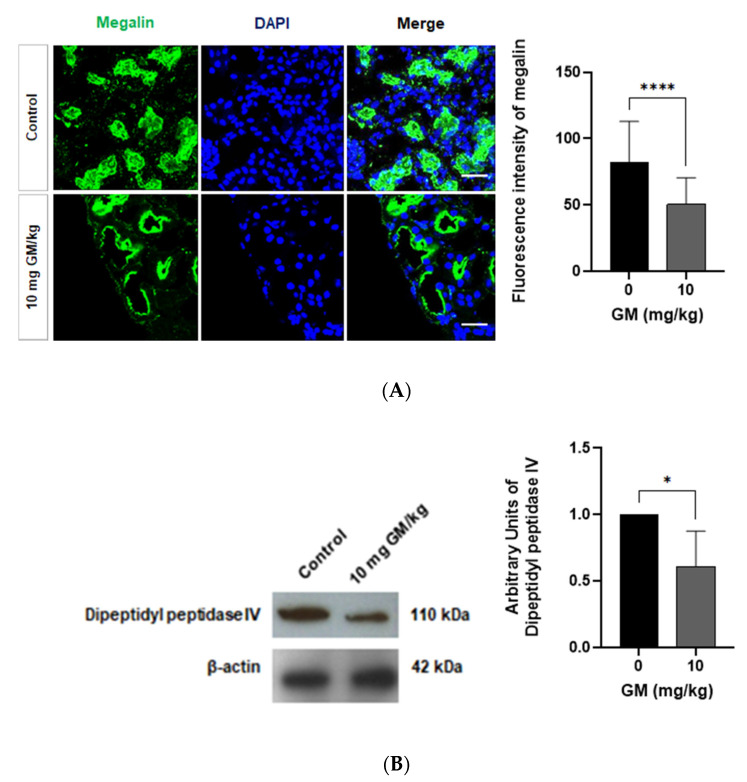
The protein expression of megalin and dipeptidyl peptidase IV decreases in PT after GM administration (**A**) Representative micrographs showing the expression of megalin in PT from untreated mice and GM exposed mice. Nuclei are stained blue with DAPI. Scale Bar = 50 µm. Bar graph shows means ± SD of a series of images from 3 different mice. Student’s *t*-test, **** *p* < 0.0001; (**B**) Representative blots of dipeptidyl peptidase IV and β-actin from whole renal tissue of untreated and GM-exposed mice. Bar graph shows means ± SD. Student’s *t*-test, * *p* < 0.05. *n* = 5 animals per dose.

A quantitative analysis of immunofluorescence labeling of megalin expressed in PT shows that the dose of 10 mg/kg of GM significantly decreased the fluorescence intensity of the megalin receptor in the PT of the treated group (Figure 3A). Also, we found that GM administration decreased the expression of other PT specific proteins, such as dipeptidyl peptidase IV that is abundantly expressed on the BBM of the S1-S3 segments of the PT [44,45] (Figure 3B).

**Figure 4:**
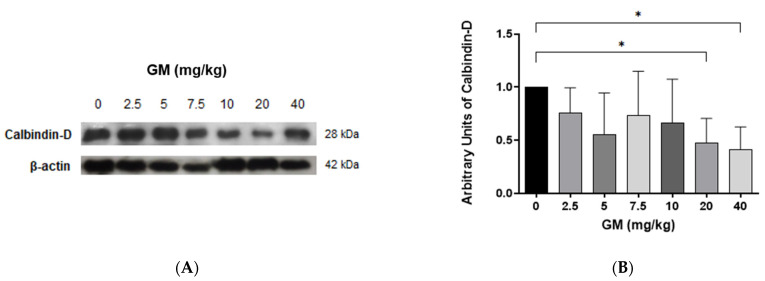
Calbindin-D protein levels in renal tissue decrease after GM administration indicating that DT injury occurs at GM doses >10 mg/kg. (**A**) Representative blot of Calbindin-D and β-actin from whole renal tissue of untreated and GM-exposed mice; (**B**) Bar graph shows means ± SD of normalized densitometries. *n* = 5 animals per dose. One-way ANOVA was performed, *p* = 0.0733. Post-hoc: Dunn test. * *p* < 0.05.

To investigate the role of nephron segments expressing 24p3R, namely DT and CD [34,35,36], in handling of CdMT in control and GM-treated animals, we focused on DT, which are located in the renal cortex and can be easily identified by the use of specific markers, for instance Calbindin-D, a vitamin D-dependent Ca^2+^-binding protein that is selectively is expressed in the DT [45,46,47]. Moreover, isolated DT can be easily separated from isolated PT by Percoll gradient centrifugation (see Methods). Importantly, in this study, we noticed that the levels of Calbindin-D decreased upon administration of doses of GM exceeding 10 mg/kg/day of GM (Figure 4), changes which could have also contributed to renal injury at higher doses.

**Figure 5:**
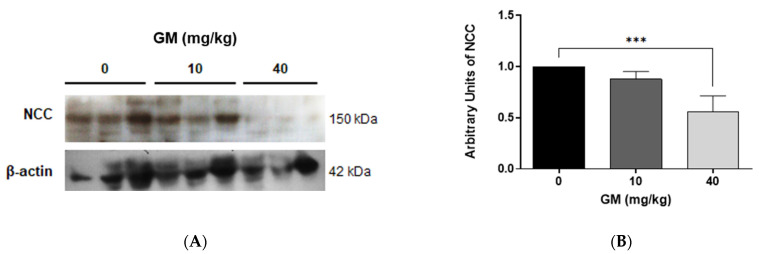
NCC protein levels in renal tissue decrease only with 40 mg/kg/day of GM. (**A**) Representative blot of NCC and β-actin from whole renal tissue of untreated and GM-exposed mice; (**B**) Bar graph shows means ± SD of normalized densitometries. *n* = 5 animals per dose. One-way ANOVA was performed, *p* < 0.0001. Post-hoc: Dunn test *** *p* < 0.005.

To further investigate the effect of 10 mg/kg versus 40 mg/kg of GM in the DT, the expression of NCC was assessed, which is expressed in the apical plasma membrane of epithelial cells of the DT and mediates the reabsorption of sodium [48]. NCC protein levels decreased with the higher dose of GM (Figure 5), indicating DT injury at 40 mg/kg GM only and confirming the data shown in Figure 4.

### 2.3. Effect of GM Administration on Protein Excretion

**Figure 6:**
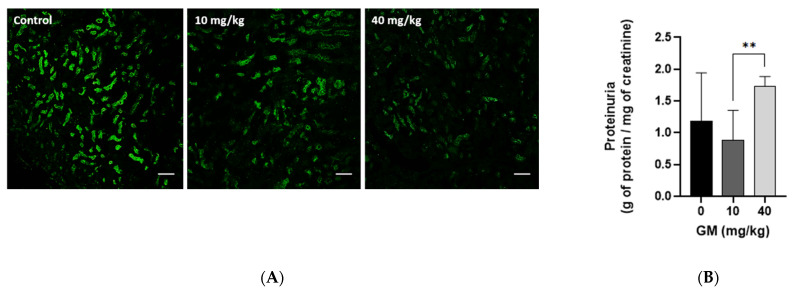
Administration of 10 mg/kg of GM decreases the levels of megalin receptor in PT but does not increase urinary excretion of proteins. (**A**) Expression pattern of megalin receptor (green fluorescence) in renal tissue after exposure to 10 and 40 mg of GM/kg/day. Scale bars represent 50 µm; (**B**) Administration of 10 mg/kg of GM does not increase urinary protein excretion of C57BL/6J mice. Bar graph shows means ± SD, *n* = 5 animals per dose. One-way ANOVA was performed, *p* = 0.0559. Post-hoc: Dunn test ** *p* < 0.025.

To assess the impact of GM-induced decrease of megalin receptor in the PT (Figure 3A) on reduction of protein endocytosis and subsequent urinary protein losses, megalin expression at 10 and 40 mg/kg GM was compared with the level of urinary protein excretion. Megalin expression was significantly reduced at 10 mg/kg GM and was almost completely absent at 40 mg/kg GM (Figure 6A). Interestingly, proteinuria was not increased after exposure to a dose of 10 mg/kg GM, despite significant reduction of megalin expression in the PT (Figure 6A). In contrast, proteinuria was significantly increased with the 40 mg/kg dose (Figure 6B), which suggests the presence of a compensatory mechanism for protein uptake downstream of the PT evident at 10 mg/kg GM. At the dose of 40 mg/kg, this compensatory effect was no longer apparent, likely due to more prominent damage and hence abolished protein uptake by the PT. Further, increased proteinuria at 40 mg/kg GM may also be the consequence of additional damage of the distal nephron, which would reduce or even prevent the compensatory mechanism of protein endocytosis observed at the lower dose of 10 mg/kg (see Figure 4 and Figure 5).

In the light of these findings, to test the hypothesis that 24p3R contributes to endocytosis of CdMT in the DT as a compensatory mechanism of protein uptake by kidneys with PT damage, subsequent experiments were performed only with the dose of 10 mg of GM/kg. The underlying rationale was that this dose causes significant PT injury, as evidenced by an increase of Kim-1 levels and a decrease of megalin and dipeptidyl peptidase IV (Figure 2, Figure 3 and Figure 6), but that the glomerulus and DT are not affected, as demonstrated by plasma creatinine, renal nephrin, Calbindin-D, and NCC levels (Figure 1, Figure 4 and Figure 5).

### 2.4. Expression of Megalin Receptor and 24p3R in the Renal Cortex of Mice Exposed to GM

**Figure 7:**
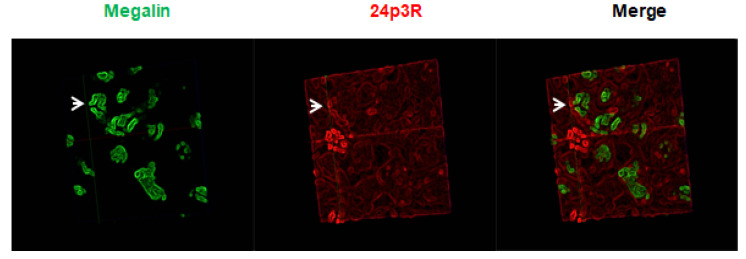
Expression of megalin in PT does not colocalize with 24p3R. Representative micrographs of the expression pattern of 24p3R (red) of kidney samples from the control group. Megalin (green): Specific protein of the PT. 40× objective. White arrows show that megalin positive cells do not express 24p3R.

Immunolabeling of renal cortex with antibodies against the C-terminal sequence of 24p3R shows that 24p3R is not colocalized with PT cells where megalin is found (Figure 7), a finding which corroborates previous experimental evidence in cultured cells and in vivo, where 24p3R was found expressed in DT and CD, but not in PT [34,35,36].

**Figure 8:**
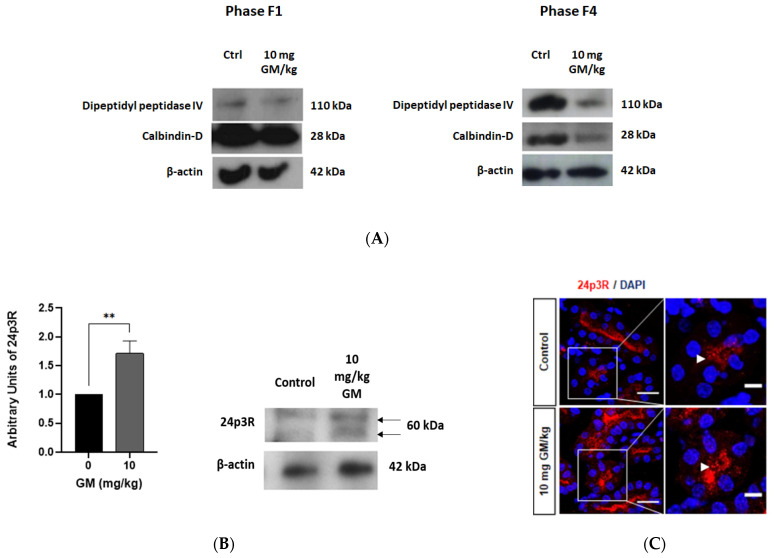
GM-induced PT injury is associated with increased 24p3R expression in DT. (**A**) Western blots of F1 (DT enriched) and F4 fractions (PT enriched) obtained after ultracentrifugation of tubules on a Percoll gradient. Dipeptidyl peptidase IV: PT marker; Calbindin-D: DT marker; (**B**) Representative blots of 24p3R and β-actin in DT enriched F1 fraction of untreated and GM-exposed mice (10 mg/kg). The bar chart shows means ± SD of normalized densitometry values. Student’s *t*-Test was performed. ** *p* < 0.05. *n* = 3. Each experiment was performed with 10 animals to obtain a pool of DT; (**C**) Representative micrographs showing the expression of 24p3R in renal tissue of control and GM-treated mice. Arrows show expression of 24p3R. Nuclei are stained with DAPI (blue). Scale Bars = 50 µm (**left**) and 20 µm (**right**).

To further establish the localization of 24p3R expression in DT but not in the PT, isolated PT (F4 phase) and DT nephron segments (F1 phase) from control and animals exposed to 10 mg/kg GM were separated by ultracentrifugation on a Percoll gradient (see Methods). Western blots of the isolated fractions were performed using antibodies against the PT- and DT-specific markers dipeptidyl peptidase IV and Calbindin-D, respectively, which confirmed enrichment of isolated PT and DT in the F4 and F1 fractions, respectively (Figure 8A). Moreover, at 10 mg/kg GM dipeptidyl peptidase IV was significantly reduced in PT (F4 fraction), whereas Calbindin-D was not changed in DT (F1 fraction), further proving selective PT damage (Figure 3, Figure 4, Figure 5 and Figure 6).

Immunoblots with an antibody against the C-terminus of 24p3R detected a double band at ~60 kDa in the F1 fraction, where DT are enriched, which is consistent with published studies with other 24p3R antibodies and suggests posttranslational modifications [34,49]. Strikingly, the levels of 24p3R protein significantly increased after GM administration (Figure 8B). To confirm this observation with a different method, immunolabeling of renal tissue was performed and again demonstrated 24p3R upregulation after PT injury induced by GM administration (Figure 8C). In contrast, immunolabeling of 24p3R was not detectable in isolated PT from control and treated groups (data not shown). Hence, these data prove selective PT damage by 10 mg/kg GM and clearly establish localization of 24p3R in DT as well as its upregulation in DT of animals exposed to 10 mg/kg GM.

### 2.5. Endocytosis of Filtered CdMT Complex by PT or DT (and Possibly CD) Expressing 24p3R in Untreated or GM-Treated Mice

**Figure 9:**
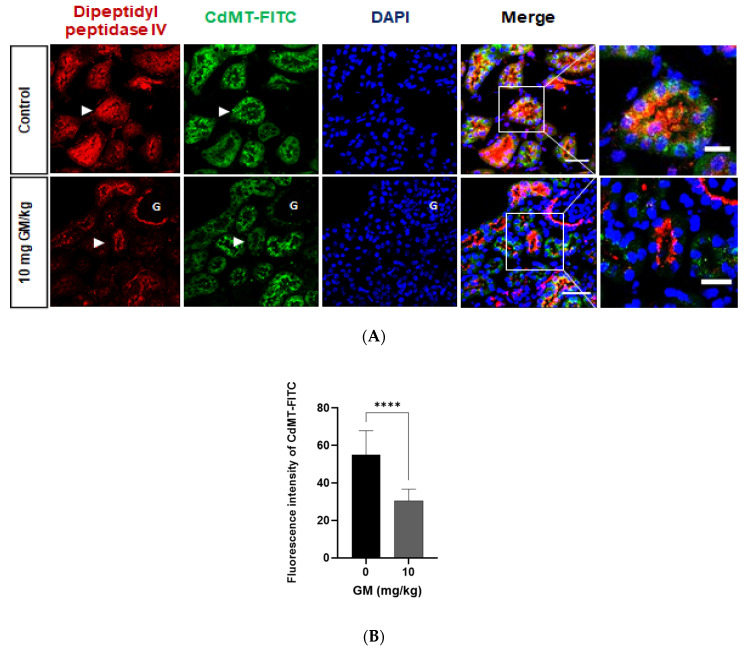
Uptake of CdMT by Dipeptidyl Peptidase IV (DPPIV) positive PT cells decrease after GM administration. (**A**) Representative micrographs of CdMT complex immunofluorescence intensity (green) with stained DPPIV (red) and nuclei (blue DAPI). Scale Bars = 50 µm (**left**) and 20 µm (**right**). G: glomerulus. White arrowheads point at magnified tubules in the merged images of control and GM treated animals. (**B**) Quantification of CdMT complex immunofluorescence intensity in DPPIV positive cells (PT). The bar graph displays means ± SD of five randomized photographs per animal (three animals per group). Student’s *t*-test was performed, **** *p* < 0.0001.

To gain more insight into the dynamics of protein uptake by PT and distal nephron segments of renal cortex expressing 24p3R (DT and CD) [34,35,36] in vivo, we injected intravenously CdMT complex coupled to FITC in C57BL/6J mice, and the animals were euthanized after 15 min. To explore if the CdMT-FITC complex is taken up by PT, co-localization of CdMT-FITC was assessed with the PT marker dipeptidyl peptidase IV. Confocal images showed that PT cells of untreated mice internalize labelled CdMT complex, which co-localized with the PT marker dipeptidyl peptidase IV. After PT damage induced by GM administration, decreased expression of dipeptidyl peptidase IV was associated with decreased CdMT uptake (Figure 9), which is the consequence of the observed decrease of megalin expression in GM-treated animals (Figure 3 and Figure 6).

**Figure 10:**
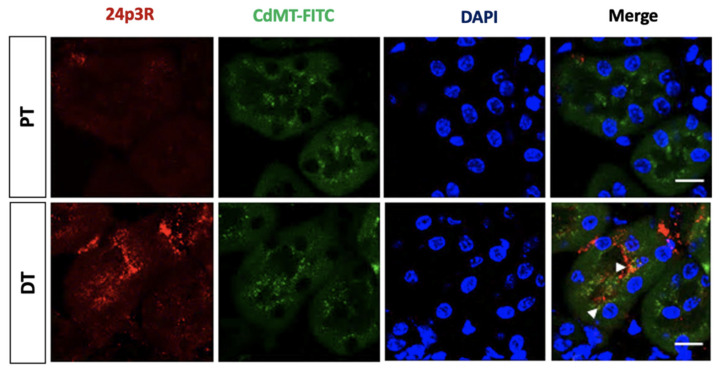
The CdMT-FITC complex is taken up by 24p3R negative PT and also co-localizes with DT cells expressing 24p3R (and possibly CD cells) in control mice. Nuclei are stained blue with DAPI. Scale Bar = 50 µm. White arrows point at the CdMT complex in 24p3R positive cells.

Moreover, after intravenous administration of the CdMT-FITC complex in control mice, CdMT-FITC was not only internalized by PT, but also co-localized with 24p3R positive cells (Figure 10) that represent DT (and possibly CD) cells (see also Figure 7 and Figure 8).

**Figure 11:**
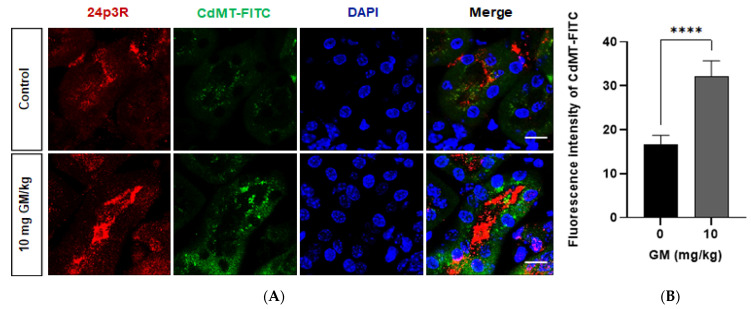
Internalization of the CdMT-FITC complex increases in renal cortex cells expressing 24p3R (DT and possibly CD) after GM exposure. (**A**) Representative micrographs of cells expressing 24p3R from untreated and GM-exposed mice. Nuclei are stained with DAPI (blue). Scale Bar = 50 µm; (**B**) Bar chart displaying means ± SD of five randomized photographs per animal (three animals per group). Quantification of CdMT complex immunofluorescence intensity (green) was performed on kidney sections stained for 24p3R (red). Student’s *t*-test was performed, **** *p* < 0.0001.

Most importantly, confocal images show that uptake of labelled CdMT complex by 24p3R positive cells in untreated mice significantly increased after GM-induced PT damage (Figure 11). CdMT uptake was increased in nephron segments with 24p3R upregulation, as observed in isolated DT (Figure 8), which is consistent with CdMT accumulation in DT (over)expressing 24p3R. No labeling was detected following the administration of uncoupled FITC.

**Figure 12:**
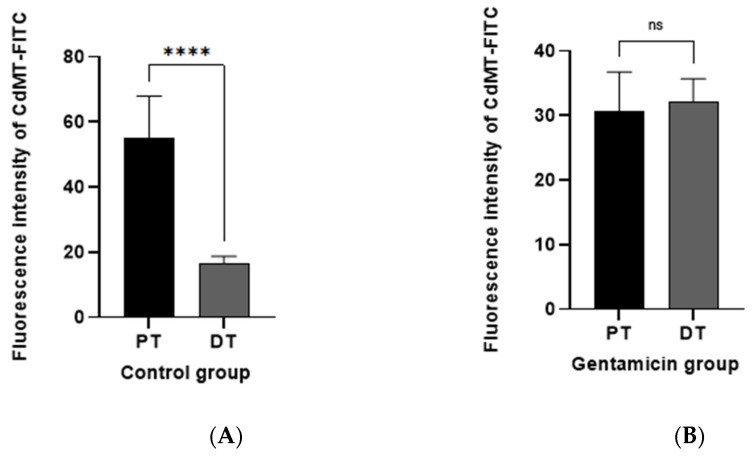
CdMT accumulates in the PT in control mice, which is compensated by the DT after PT damage induced by 10mg/kg gentamicin. Bar chart showing means ± SD of five randomized photographs per animal (three animals per group) Quantification of CdMT complex immunofluorescence intensity (green) was performed on kidney sections. Student’s *t*-test was performed, (**A**) Control; **** *p* < 0.0001; (**B**) GM; *p* = 0.6769. ns = not significant.

In addition, quantitative analysis of CdMT uptake showed that the CdMT complex in control animals is mainly internalized by nephron segments not expressing 24p3R, namely PT (Figure 12A) (see also Figure 10). After damage induced by 10 mg/kg GM, uptake was reduced in PT but increased in 24p3R positive DT cells (and possibly CD) (Figure 12B), further supporting the hypothesis that CdMT uptake in the DT compensates for loss of uptake capacity in the PT, and is associated with upregulation of 24p3R (see Figure 8).

## 3. Discussion

In this study, we evaluated the endocytosis of the CdMT complex by 24p3R positive cells in distal segments of the nephron after PT damage. Our main finding is that PT damage caused by GM administration increases 24p3R protein expression in DT cells (Figure 8B). In rodents, 24p3R is not expressed in PT cells where megalin is localized (see Figure 7), but in the DT (and possibly CD) (Figure 8 and [34,35,36]). Our in vivo study significantly extends these previous reports functionally and toxicologically by demonstrating that in GM-treated animals with PT damage upregulation of 24p3R increases the uptake of the CdMT complex in the distal segment of the nephron, most likely as a compensatory mechanism for defective protein endocytosis in the PT.

We used GM as an in vivo model of PT injury [40,41,42], which is caused by the deleterious effect of GM on the BBM, as evidenced by a decrease of dipeptidyl peptidase IV, a specific protein of BBM (Figure 3, Figure 8 and Figure 9). These results are consistent with previously reported damaging renal effects induced by aminoglycosides, which include structural changes and functional impairment of the plasma membrane, mitochondria, and lysosomes in PT cells [50,51,52].

Although GM at different doses did not modify glomerular function, endocytosis of GM in PT cells led to PT damage, as evidenced by an increase in the expression of the sensitive renal biomarker Kim-1 (see below) (Figure 2). This is in agreement with a previous dose-response study with rats that were subcutaneously exposed to GM for 3 days [53]. This study showed that while the biomarkers of kidney damage BUN (blood urea nitrogen), serum creatinine and NAG (N-acetyl-D-glucosaminidase) only increased with the highest dose (400 mg/kg), urinary levels and mRNA expression of Kim-1 in kidney tissue increased in a dose-dependent manner after the administration of GM already at a dose ≥100 mg/kg, a pattern that was also observed after administration of compounds such as mercury and chromium [53]. Kim-1, a transmembrane glycoprotein with an extracellular immunoglobulin-like domain and a long mucin-like domain [54], is expressed at low levels in the normal kidney. Most importantly, Kim-1 is upregulated more than any other protein in the PT during kidney injury, such as ischemia/reperfusion injury or drug-induced AKI, and used as a sensitive marker for renal injury, including nephrotoxic animal models [54,55,56,57,58,59]. However, when Kim-1 is expressed chronically, it results in progressive kidney fibrosis and chronic kidney failure [60].

GM is also taken up by DT through an endocytosis-independent mechanism, such as the non-selective cation channel TRVP [61,62], and may therefore also cause cytotoxicity of this segment. consequently, the expression of Calbindin-D, an intracellular Ca^2+^-binding protein that is exclusively localized in DT [45,46,47] and the thiazide-sensitive NaCl cotransporter NCC, which mediates the reabsorption of sodium in the DT [48], were evaluated to test the effect of GM on DT (Figure 4 and Figure 5). Our results demonstrate that a dose of 10 mg of GM/kg/day caused PT injury, as indicated by an increase of Kim-1 levels and by decreased dipeptidyl peptidase IV and megalin expression (Figure 2, Figure 3, Figure 6, Figure 8 and Figure 9), whereas the levels of Calbindin-D and NCC in DT were not affected (Figure 4 and Figure 5). However, the highest dose of GM (40 mg/kg/day) did decrease the levels of Calbindin-D and NCC (Figure 4 and Figure 5). Calbindin-D modulates Ca^2+^ transport by facilitating its reabsorption through the cell [63]. Although we did not evaluate Ca^2+^ excretion after GM administration, intracellular movement of Ca^2+^ ions directly depends on the cellular concentration of Calbindin-D. Therefore our data suggest that the reduction of Calbindin-D protein levels induced by high GM doses could cause a decrease in Ca^2+^ reabsorption, increasing its excretion in the urine [64]. Indeed, increased urinary fractional excretion of Na^2+^, K^+^, Ca^2+^ and Mg^2+^ has been reported in rats treated with 40 mg/kg GM [65].

Experimental evidence has demonstrated that physiologically a small but significant proportion of filtered proteins is reabsorbed by the distal segments of the nephron, particularly after glomerular or PT damage, where distal segments may become more relevant for membrane transport, and in particular protein uptake [22]. When CdMT was administered intravenously, 24p3R positive cells in DT (and possibly CD) internalized the fluorescence-labelled complex in the control group (Figure 10). This uptake was significantly increased after PT damage (Figure 11), consistent with the increased expression of 24p3R in isolated DT after GM exposure (Figure 8). We propose that 24p3R upregulation acts as a compensatory mechanism for protein endocytosis when PT function is defective, e.g., in the experimental model of GM-induced damage, or following hereditary or acquired Fanconi syndrome [23,24,25]. With a 10 mg/kg dose of GM, urinary excretion of proteins did not change, despite reduced megalin expression in the PT (Figure 3, Figure 6 and Figure 9), whereas at the dose of 40 mg/kg GM this compensatory effect was no longer evident (Figure 6B). We interpret these data by proposing the presence of two mechanisms for RME of proteins, the first one located in PT and the second in the distal nephron (DT and CD). Under physiological conditions, a high-capacity and low-affinity receptor for filtered protein in the PT mediates bulk uptake of filtered proteins, namely megalin:cubilin [10,11]. Proteins that escape PT endocytosis, either due to their low concentration in the primary filtrate or because of receptor saturation, are taken up by a high-affinity and low capacity receptor in the DT and CD, namely the 24p3R [34,66]. In the healthy kidney, both protein uptake systems prevent proteinuria. Partial damage of the PT mechanism of protein endocytosis, which is mimicked by the GM dose of 10 mg/kg (Figure 3, Figure 6 and Figure 9), partly compromises PT protein endocytosis and thus allows more filtered proteins to escape into the distal nephron. However, these ‘leaked” proteins are recaptured by RME via 24p3R in the DT and CD. Moreover, this compensatory protein reuptake also involves upregulation of the 24p3R receptor (Figure 8). Complete disruption of the PT functions, however, leads to massive proteinuria (Figure 6B) because the high-affinity low-capacity receptor 24p3R in the distal nephron quickly saturates. In our experimental model, 40 mg/kg GM did not only cause major disruption of PT function, as evidenced by an almost complete absence of megalin expression (see Figure 6), but also partly disrupted DT function (Figure 4 and Figure 5), which may also have contributed to increased proteinuria.

When high micromolar concentrations of labelled CdMT are injected into experimental animals, as in the present study, the plasma concentration of CdMT largely exceeds the physiological concentration (the plasma CdMT concentration in the present study was ~20 μmol/L) [3,27]. It is therefore not surprising that fluorescence-labelled CdMT was internalized by PT cells in the control group considering a *K_D_* of megalin for MT of ~100 µM) [26]. However, after GM administration, endocytosis of CdMT complex in PT decreased, as evidenced by decreased labeling of dipeptidyl peptidase IV positive cells (Figure 9A) indicating the reduction of BBM and megalin receptor expression (Figure 3 and Figure 6). Experimental evidence shows that Cd-β2-microalbumin, Cd-albumin and Cd-Lcn2 are taken up by PT cells via megalin:cubilin and cause toxicity at ultrafiltrate concentrations [28]. They therefore represent more plausible candidates of Cd-protein complexes that damage the renal PT via megalin:cubilin dependent endocytosis. For this reason, CdMT and other Cd-protein complexes that are not bound by megalin could bypass endocytosis in the PT and reach the distal nephron posing a risk at that segment of the nephron as well (reviewed in [3]).

Recent evidence shows that uptake of Fe in the distal nephron also involves RME of Fe-protein complexes via 24p3R [34,39]. These cell culture studies demonstrated that 24p3R has a high affinity for Tf and hemoglobin, but also albumin and MT, all ligands of the megalin receptor in PT [10,11]. Because MT, Tf and albumin do form complexes with various transition metals, including Fe and Cd^2+^ [28], 24p3R also internalizes metal-protein complexes such as the CdMT complex [34], which is supported by the current findings (Figure 8, Figure 9, Figure 10, Figure 11 and Figure 12). CdMT is the predominant form in which Cd^2+^ is found in the circulation after Cd^2+^ exposure and redistribution to the kidney [7,8].

The proposed hypothesis of two sequential receptors for protein endocytosis in the nephron (megalin:cubilin in the PT and 24p3R in DT and CD) with different binding affinities and uptake capacities is further supported by an in vivo study with mutant Lcn2/24p3 [67]. Circulating 24p3 is filtered by the glomeruli and 60–70% is reabsorbed from the lumen by the PT megalin receptor in the PT, where it is degraded [68]. Interestingly, a mutant form of 24p3 was designed that could bypass megalin-dependent PT endocytosis in an in vivo animal model, and trace amounts of the mutant protein were reabsorbed by principal and intercalated cells of the renal medulla where 24p3R is expressed [34,35,36], proving megalin-independent 24p3 uptake in the distal [67]. Strikingly, we have previously shown that 24p3 is endocytosed by cultured DT and CD cells via high-affinity binding to 24p3R [34,66]. Hence, the high-affinity protein receptor 24p3R in DT and CD likely contributes to protein endocytosis and depletes the final urine from proteins under physiological conditions or limits losses associated with renal diseases, including various forms of inherited or acquired Fanconi syndrome [23,24,25].

What could be the signaling mechanism underlying 24p3R upregulation? Albumin internalization by 24p3R can activate NF-κB and TGF-β1 signaling pathways, associated with proinflammatory and profibrotic mechanisms in response to proteinuria [35]. But inflammatory signaling reduces 24p3R expression in renal cells [69], possibly via NF-κB activation [70], which however would not explain 24p3R upregulation in DT observed after exposure to 10 mg/kg GM (Figure 8). Other mechanisms of transcriptional upregulation of 24p3R have been described in various experimental models (including renal cells), such as Runx3 [71] or NFAT5/TonEBP [70]. Interestingly, we have observed a similar upregulation of 24p3R in the renal cortex and medulla of kidney-specific megalin:cubilin-deficient mice [72] (F. Thévenod, Wing-Kee Lee & Rikke Nielsen; unpublished observations). These data suggest that upregulation of 24p3R in the distal nephron after disruption of PT protein endocytosis may be a more general phenomenon. Upregulation of 24p3R associated with proteinuria may occur independently of Cd^2+^ nephrotoxicity and involve a signaling cross-talk between proximal and distal nephron segments. However, the mechanisms underlying 24p3R upregulation described in this study remain to be investigated and will be the focus of future studies.

## 4. Materials and Methods

### 4.1. Antibodies

The following antibodies were used in this work: anti-SLC22A17/24p3R (cat. No. PAB13044; ABNOVA, Taipei, Taiwan), anti-Calbindin-D 28K (cat. No. 711443; ThermoFisher Scientific, Waltham, MA, USA), anti-thiazide sensitive NaCl cotransporter (NCC) (cat. No. AB3553, Merck Millipore, Burlington, MA, USA), anti-CD26 or Dipeptidyl peptidase IV (cat. No. AB28340; ABCAM, Cambridge, UK), anti-megalin (cat. No. sc-16478; Santa Cruz Biotechnology, Dallas, TX, USA), anti-TIM-1 or Kim-1 (cat. No. PA5-20244; ThermoFisher Scientific, Waltham, MA, USA), anti- β-actin (cat. No. SC-4778; Santa Cruz Biotechnology, Dallas, TX, USA), anti-mouse (cat. No. C02-7076S; Cell Signaling, Danvers, MA, USA), anti-rabbit (cat. No. sc-2004; Santa Cruz Biotechnology, Dallas, TX, USA).

### 4.2. Animals

The Institutional Committee for the Care and Use of Laboratory Animals (Comité Interno para el Cuidado y uso de los Animales de Laboratorio, CICUAL) from CINVESTAV approved all animal procedures (protocol number: 0188-16). All experimental procedures were conducted according to the current Mexican legislation NOM-062-ZOO-1999 (SAGARPA) and in agreement with the Guide for the Care and Use of Laboratory Animals of the National Institutes of Health (NIH).

C57BL/6J male mice (8–12 weeks) were housed in polypropylene cages with sawdust bedding, located in a room with 12/12 h light/dark cycles, at 22 ± 2 °C and 50 ± 5% humidity. Animals had *ad libitum* access to food and water. To induce damage in the PT, animals were subcutaneously administered daily with different doses of the aminoglycoside GM for 7 days. Aminoglycosides are low-protein binding drugs that are not metabolized in the body and are freely filtered through the glomerular filtration barrier, being reabsorbed and accumulated in PT cells [40] by the multiligand endocytic receptor megalin:cubilin [41]. The doses of GM used were 2.5, 5, 7.5, 10, 20 and 40 mg/kg/day diluted in isotonic saline solution. A control group was subcutaneously administered with an isotonic saline solution. One day after the end of the treatment, mice were housed individually in metabolic cages to collect 16 h urine. After urine collection, mice were anesthetized with isoflurane (cat No. Q-7833-222 Sofloran; Vet Pisa Farmacéutica, Hidalgo, Mexico) using V-1 Tabletop Lab Animal System (VetEquip, Inc., Pleasanton, CA, USA) and blood was collected by cardiac puncture (terminal exsanguination) with a heparinized syringe and the blood was collected in heparinized capillary tubes. Blood samples were centrifuged at 2500× *g* for 15 min at 4 °C to obtain samples. Urine samples were centrifuged for 10 min at 1000× *g* and aliquots were separated. All samples were stored at −70 °C until use.

After exsanguination, kidneys were perfused with isotonic saline solution via the renal artery before they were extracted and washed in cold phosphate-buffered saline (PBS). One kidney was immersion-fixed in 4% paraformaldehyde in PBS (PFA-PBS) overnight at 4 °C, and the second kidney was stored at −70 °C until use.

The isolation and purification of mouse DT was based on the method of Vinay et al. [73]. Once the kidneys were obtained, they were placed in cold Krebs-Henseleit Solution (KHS) pH 7.4 previously gassed with 95% O_2_/5% CO_2_. After decapsulation, the cortex was dissected and the slices were transferred into a flask with 10 mL of KHS,15 mg of collagenase (from *Clostridium histolyticum*, type II) and 0.5 mL of 10% of bovine serum albumin (BSA) (Equitech-Bio Inc., Kerrville, TX, USA). Samples were gassed with 95% O_2_/5% CO_2_ for 30 min at 37 °C with constant agitation. After digestion, 30 mL of ice-cold KHS with a protease inhibitor cocktail (Complete; Roche Diagnostics, Indianapolis, IN, USA) were added, and the suspension was gently agitated to disperse tissue fragments. Suspension was filtered through a tea strainer to remove the collagen fibers and the tissue suspension was gently centrifuged at 60× *g* for 30 s. The pellet was resuspended in 10 mL of ice-cold KHS with the protease inhibitor cocktail. This washing procedure was repeated three times. After the last wash, the pellet was resuspended in 5% BSA solution with protease inhibitors for 5 min at 4 °C. The suspension was gently centrifuged at 60× *g* for 30 s and the supernatant was discarded. Tissue pellets were suspended in 30 mL of a freshly prepared mixture of ice-cold Percoll (cat. No. 1002281423; Sigma Aldrich, St. Louis, MO, USA) and KHS (1:1, *v*/*v*). Thereafter, the suspension was centrifuged at 12,200× *g* for 30 min at 4 °C, resulting in a separation of four bands. The first band was enriched with DT, the second and the third band have a mixture of nephron segments that include glomeruli, proximal and distal segments and the fourth band (deepest) was enriched in PT. Separation of the nephron segments PT and DT was confirmed by Western blotting for dipeptidyl peptidase IV and Calbindin-D, respectively.

### 4.3. Urine and Plasma Measurements

Total urinary protein was measured using the Bradford method with the Quick Start Bradford Protein assay (cat. No. 500-0205; Bio-Rad Laboratories, Hercules, CA, USA). Plasma and urinary creatinine were measured using a commercial kit (cat. No. CR510; Randox Laboratories, Crumlin, UK) based on the Jaffe method. All tests were performed in triplicate.

### 4.4. Magnetic Luminex Assay

Renal tissue samples were employed to quantify kidney injury biomarkers, nephrin and Kim-1, using the Magnetic Luminex Assay Mouse Premixed Multi-Analyte Kit (cat. No. LXSAMSM-03; R&D Systems, Minneapolis, MN, USA). Manufacturer’s instructions were followed. All samples were analyzed in duplicate. The plate was read on a Magpix^®^ System (Millipore Corp., Burlington, MA, USA).

### 4.5. Western-Blot Assay

Protein extraction for the detection of Calbindin-D, NCC, dipeptidyl peptidase IV or 24p3R, was performed using a lysis buffer that contained protease inhibitors (Complete; Roche Diagnostics, Indianapolis, IN, USA). Afterwards, the samples were centrifuged at 4 °C for 30 min at 13,000× *g*. Protein concentration of the supernatants were determined using the Bradford method with the Quick Start Bradford Protein assay (cat. No. 500-0205; Bio-Rad Laboratories, Hercules, CA, USA). Total protein (50 µg) was loaded for each sample on 10% SDS-PAGE gels. Gels were run at 100 V. Also, a molecular weight standard (cat. No. 1610374; Precision Plus Protein Bio-Rad, Laboratories Inc., Hercules, CA, USA) was run in parallel to corroborate the molecular weight of the proteins of interest. Proteins were transferred for 1 h at 20 V in semi-dry transfer onto polyvinylidene fluoride membranes (cat. No. 162-0177; Bio-Rad, Laboratories Inc., Hercules, CA, USA). Nonspecific protein binding was blocked with 5% low-fat milk and 1% BSA for 1 h at room temperature. Membranes were incubated overnight at 4 °C with the appropriate primary antibodies anti-SLC22A17/24p3R (dil. 1:250), dipeptidyl peptidase IV (dil. 1:1000), Calbindin-D (dil. 1:1000) or NCC (dil. 1:500). Thereafter, membranes were incubated with peroxidase-conjugated secondary antibodies anti-mouse or anti-rabbit (dil. 1:1000) for 2 h at room temperature. Immunoblots were developed using autoradiographic plaques and Luminata Forte Western HRP substrate (cat. No. WLBUF0500, Millipore, Burlington, MA, USA). Quantification of immunopositive bands was performed with the ImageJ software, and signal intensities were normalized to actin levels. All tests were performed in triplicate.

### 4.6. Conjugation of CdMT to Fluorescein Isothiocyanate (FITC)

Cd^2+^ coupled to MT was prepared as previously described [74]. Briefly, apo-MT was prepared by acidification of commercially available MT-1 (cat. No. COP03; Creative BioMart, New York, NY, USA) in 0.1 N HCl followed by filtration, equilibrated with 0.01 N HCl. The apo-MT was mixed with a 10-fold molar excess solution of cadmium chloride (CdCl_2_) (Sigma-Aldrich Co., St. Louis, MO, USA). The unbound metal was removed by filtration with an Amicon centrifugal filter unit according to the manufacturer’s instructions (cat. No. 500324; Amicon Ultra-0.5 Centrifugal Unit Millipore, Burlington, MA, USA). Subsequently, 0.01N HCl was added. Diluent was added to reach a final concentration of 1 mg CdMT/mL.

Conjugation of the CdMT complex with FITC was performed according to the manufacturer’s instructions (cat. No. F6434, FluoReporter^®^ FITC Protein Labeling Kit, Invitrogen, Waltham, MA, USA). Briefly, the CdMT solution was mixed with a 1M sodium bicarbonate solution and a 10 mg/mL reactive dye stock solution at room temperature for 1 h, protected from light. To purify the CdMT-FITC complex, Micro Bio-SpinTM Chromatography columns were used (cat. No. 7326200; Micro Bio-SpinTM P-6 Gel Columns, SSC Buffer, Bio-Rad, Hercules, CA, USA). Once the buffer of the columns was drained, the sample was placed directly to the center of the column and was centrifuged for 4 min at 1000× *g*. Following centrifugation, the purified samples were obtained and stored a −4°C, protected from light until use.

### 4.7. Injection of the CdMT FITC Complex

One day after the end of the treatment, control and gentamicin group mice (10 mg/kg/day) were anesthetized with isoflurane (cat No. Q-7833-222 Sofloran; Vet Pisa Farmacéutica, Hidalgo, Mexico) using V-1 Tabletop Lab Animal System (VetEquip, Inc., Pleasanton, CA, USA) and were injected intravenously with 10 µg/g body weight of CdMT-FITC complex diluted in phosphate-buffered saline (PBS) 1×, and euthanized 15 min after the injection, by retrograde aortic perfusion with saline solution. Kidneys were obtained and used for the immunofluorescence assay (See the “Immunofluorescence” section). Immunofluorescence images of renal tissue were used to determine the level of uptake of the CdMT-FITC complex in the nephron. Dipeptidyl peptidase IV was used as a marker of PT cells and the anti-SLC22A17/24p3R was used as a marker of DT cells.

### 4.8. Immunofluorescence

Following animal euthanasia, renal tissue was immersion-fixed in 4% paraformaldehyde in PBS (PFA-PBS) overnight at 4 °C. Afterwards, kidney sections were incubated in a 30% sucrose solution overnight. Kidney slices of 8 µm thickness were obtained with a Leica cryostat and mounted on gelatin-coated slides that were kept frozen at −70 °C until use. Renal tissue was permeabilized with 0.2% Triton X-100 in PBS for 30 min and incubated in a blocking solution containing 1% BSA diluted in 0.2% PBS-Triton X-100, for 1 h at room temperature. The slides were incubated with the following primary antibodies at 4 °C, overnight: anti-SLC22A17 (dil. 1:150), anti-megalin (dil. 1:50), anti-dipeptidyl peptidase IV (dil. 1:150), anti-Calbindin-D (dil. 1:150) and anti-Kim-1 (dil. 1:50). Secondary antibodies, anti-rabbit Alexa 594 (dil. 1:600) and anti-mouse Alexa 488 (dil. 1:600), were incubated for 2 h at room temperature. Confocal images were captured with a Leica SP8 confocal microscope (TCS-SP8 Leica, Heidelberg, Germany) and the images were evaluated with the Leica LAS AF Lite software, version 2.3.0.

### 4.9. Staining Quantification

Quantification of the immunofluorescence intensity of the labeled CdMT complex was performed on kidney sections co-stained for anti-SLC22A17/24p3R to distinguish DT cells (CdMT positive-24p3R positive) and dipeptidyl peptidase IV to distinguish PT cells (CdMT positive-dipeptidyl peptidase IV positive). A group of pictures (at least 5) from at least 3 different mice were acquired using a Leica SP8 confocal microscope (TCS-SP8 Leica, Heidelberg, Germany). Acquisition parameters were kept unchanged to ensure the comparability of the pictures. Using the Leica LAS AF Lite software, version 2.3.0, all the CdMT positive-24p3R positive and the CdMT positive-dipeptidyl peptidase IV positive cells were selected, and the intensity of fluorescence was quantified.

### 4.10. Statistics

Data are presented as the average ± standard deviation (SD). A one-way analysis of variance (ANOVA) was used for comparisons among multiple groups and post hoc test were performed. Comparisons between the results from the isolated DT from the control group and the GM group (10 mg/kg) were analyzed with a Student’s *t*-test. *p* < 0.05 was considered significant in all cases. All tests were performed with the GraphPad software 7.03 for Windows.

## 5. Conclusions

In this in vivo study in mice, we conclusively show that the CdMT complex is internalized by 24p3R positive cells in DT (and possibly CD) cells under physiological conditions, but even more after PT damage induced by GM. Our results strongly suggest that protein endocytosis by 24p3R becomes more relevant for protein uptake in the distal nephron to compensate for PT damage and involves 24p3R upregulation. Finally, because 24p3R can internalize CdMT complexes, our findings suggest that accumulation of CdMT in DT and CD can contribute to aggravation of renal injury and nephropathies after Cd^2+^ exposure, but also in other proteinuric renal diseases.

## Data Availability

The data presented in this study are available on request from the corresponding author.
